# Genomic and transcriptomic features between primary and paired metastatic fumarate hydratase–deficient renal cell carcinoma

**DOI:** 10.1186/s13073-023-01182-7

**Published:** 2023-05-02

**Authors:** Jiayu Liang, Guangxi Sun, Xiuyi Pan, Mengni Zhang, Pengfei Shen, Sha Zhu, Jinge Zhao, Linmao Zheng, Junjie Zhao, Yuntian Chen, Xiaoxue Yin, Junru Chen, Xu Hu, Yuhao Zeng, Jianhui Chen, Yongquan Wang, Zhihong Liu, Jin Yao, Minggang Su, Rui Huang, Banghua Liao, Qiang Wei, Xiang Li, Qiao Zhou, Jiyan Liu, Yali Shen, Zhenhua Liu, Ni Chen, Hao Zeng, Xingming Zhang

**Affiliations:** 1 Department of Urology, Institute of Urology, West China Hospital, Sichuan University, Chengdu, 610041 China; 2 Department of Pathology, West China Hospital, Sichuan University, Chengdu, 610041 China; 3 Department of Radiology, West China Hospital, Sichuan University, Chengdu, 610041 China; 4 Department of Urology, Fujian Medical University Union Hospital, Fuzhou, China; 5 Department of Urology, Southwest Hospital, Army Medical University, Chongqing, China; 6 Department of Nuclear Medicine, West China Hospital, Sichuan University, Chengdu, 610041 China; 7 Department of Biotherapy, West China Hospital, Sichuan University, Chengdu, 610041 China

**Keywords:** Fumarate hydratase–deficient renal cell carcinoma, Whole-exome sequencing, DNA methylation, RNA-seq, Tumor evolution, Metastatic lesions

## Abstract

**Background:**

Fumarate hydratase–deficient renal cell carcinoma (FH-RCC) is a rare highly aggressive subtype of kidney cancer for which the distinct genomic, transcriptomic, and evolutionary relationships between metastatic and primary lesions are still unclear.

**Methods:**

In this study, whole-exome, RNA-seq, and DNA methylation sequencing were performed on primary-metastatic paired specimens from 19 FH-RCC cases, including 23 primary and 35 matched metastatic lesions. Phylogenetic and clonal evolutionary analyses were used to investigate the evolutionary characteristics of FH-RCC. Transcriptomic analyses, immunohistochemistry, and multiple immunofluorescence experiments were performed to identify the tumor microenvironmental features of metastatic lesions.

**Results:**

Paired primary and metastatic lesions generally showed similar characteristics of tumor mutation burden, tumor neoantigen burden, microsatellite instability score, CNV burden, and genome instability index. Notably, we identified an FH-mutated founding MRCA (the most recent common ancestor) clone that dominated the early evolutionary trajectories in FH-RCC. Although both primary and metastatic lesions manifested high immunogenicity, metastatic lesions exhibited higher enrichment of T effector cells and immune-related chemokines, together with upregulation of PD-L1, TIGIT, and BTLA. In addition, we found that concurrent *NF2* mutation may be associated with bone metastasis and upregulation of cell cycle signature in metastatic lesions. Furthermore, although in FH-RCC metastatic lesions in general shared similar CpG island methylator phenotype with primary lesions, we found metastatic lesions displaying hypomethylated chemokine and immune checkpoints related genomic loci.

**Conclusions:**

Overall, our study demonstrated the genomic, epigenomic, and transcriptomic features of metastatic lesions in FH-RCC and revealed their early evolutionary trajectory. These results provided multi-omics evidence portraying the progression of FH-RCC.

**Supplementary Information:**

The online version contains supplementary material available at 10.1186/s13073-023-01182-7.

## Background

Fumarate hydratase–deficient renal cell carcinoma (FH-RCC) is a rare subtype of kidney cancer, characterized by either somatic or germline aberration of *fumarate hydratase (FH)* gene, in which pathogenic germline mutation of *FH* gene is associated with hereditary leiomyomatosis renal cell carcinoma (HLRCC) syndrome [1, 2]. FH-RCC exhibits highly aggressive clinical behaviors, with 56–63% of cases presenting with metastatic diseases at initial diagnosis and showing poor prognosis [3, 4]. Unfortunately, few effective treatment strategies are available for FH-RCC patients to date due to its rarity and our limited understanding of its molecular basis.

Previous studies have reported several mechanisms that may contribute to the aggressiveness of FH-RCC, including the Warburg effect, epithelial-to-mesenchymal transition (EMT), and CpG island methylator phenotype (CIMP) [5–9]. We and others also have delineated the clinicopathological, genomic, and epigenomic features for FH-RCC using primary tumor samples, in which we noticed the early presence of lymph node and/or bone metastasis [3, 4]. However, it is not fully understood whether the metastases in FH-RCC are molecularly similar or distinct from the primary tumors, and what are the evolutionary patterns of these metastases. Therefore, this study aims to identify the heterogeneity between matched metastatic and primary lesions of FH-RCC, reveal the evolutionary trajectory, and explore potential therapeutic strategies.

## Methods

### Patient selection and sample preparation

From 2014 to 2022, 90 cases with FH-RCC were included in our multi-center database established in West China Hospital. Generally, cases were from 19 provinces or municipalities of China. Firstly, all candidate RCC cases were screened using immunohistochemical staining (IHC) for FH protein and S-(2-succino)-cysteine (2SC) and reviewed by two experienced uropathologists. Diagnosis of FH-RCC was then confirmed by DNA sequencing with germline or somatic FH mutations.

All the FH-RCC cases in our database were systemically reviewed and 19 cases with available formalin-fixed paraffin-embedded (FFPE) surgical samples from matched adjacent normal kidney tissues, primary tumor tissues, and metastatic tissues were then selected. Finally, 19 cases with 23 primary and 35 matched metastatic lesions were selected. Whole-exome sequencing in all 58 tumor samples, Methyl-Seq and RNA-seq were performed in 37 tumor samples and 32 tumor samples, respectively. Among them, different regions of primary tumor were sampled for cases FH16, FH26, FH32, and FH42. The metastatic samples were originated from lymph node, primary surgical site, tumor thrombus, abdominal wall, retroperitoneal site, peritoneal site, ovarian, and adrenal metastasis. For each case, the matched adjacent normal tissues (*n* = 8) or blood samples (*n* = 11) were collected for whole-exome sequencing (WES) to assess the genomic mutation characteristics. The study was conducted in accordance with the Declaration of Helsinki and all clinical samples were acquired with written informed consents under permission from the Ethics Committee of West China Hospital of Sichuan University. All patients provided written consent for genetic analysis.

### Clinicopathological characteristics and outcomes

Clinicopathological data including age, gender, family history, metastatic sites, TNM stage, histological type, ISUP grade, surgery types, and systemic treatment types were retrospectively collected. Synchronous metastasis was defined as metastasis at the diagnosis of the primary renal cell carcinoma. Metachronous metastasis was defined as the presence of metastasis after a period of 3 months post resection of primary lesions. For patients receiving systemic treatments, the first-line progression-free survival (PFS) was defined as the time from treatment initiation receiving first-line systemic treatment to disease progression or death. Tumor response was defined by Response Evaluation Criteria in Solid Tumors (RECIST) version 1.1 28. For validation of the value of local treatment in patients with systemic therapy, we screened 58 patients from the previous described database. Baseline characteristics are summarized in Additional file 1: Table S1 and Additional file 2: Table S2. The modalities of systemic treatment included tyrosine kinase inhibitor (TKI) monotherapy, anti-VEGF inhibitor plus TKI, mTOR inhibitor plus TKI, mTOR inhibitor monotherapy, chemotherapy, anti-PD-1 inhibitor plus TKI, and anti-PD-1 inhibitor monotherapy. Different systemic treatments were classified into two groups: immune checkpoint blockade (ICB)-based treatment (anti-PD-1 inhibitor + TKI or anti-PD-1 inhibitor monotherapy) and not ICB-based treatment (the rest modalities of systemic treatment).

### DNA extraction

All primary and metastatic tumor sections were reviewed by two pathologists to ensure tumor sections with at least 70% tumor nuclei. Representative sections of formalin-fixed paraffin-embedded (FFPE) tumor (8 μm) and matched normal tissues/blood samples were collected. High-quality genomic DNA was extracted by using the GeneRead DNA FFPE Kit (180,134, QIAGEN, Hilden, GER) according to the manufacturer’s instructions. Germline DNA (gDNA) was extracted from white blood cells using the Blood Genomic DNA Mini Kit (CW2087, Cwbiotech, Beijing, China).

### Whole-exome sequencing

Exome capture was performed using xGen Exome Research Panel v1.0 (IDT), and this was followed by paired-end sequencing using Illumina Hiseq Xten platform (Illumine Inc, CA, USA). Mean sequencing depth was 278X (range from 36 to 576X).

### Read alignment, BAM file generation and post-alignment optimization

Clean reads were aligned to the reference human genome hg19 (Genome Reference Consortium GRCh37) using BWA 0.7.17 (Burrows-Wheeler Aligner) MEM algorithm with default parameters. BAM was coordinate sorted and PCR duplicates were removed with Sambamba version 0.6.8. After the initial alignment of WES data, we followed GATK v3.8 Best Practice to process all BAMs from the same patient. The detailed process was described in our previous studies [4, 10].

### Somatic mutations analysis and somatic mutation signature profiling

The GATK MuTect2 pipeline was run for paired tumor-normal somatic mutation calling. The resulting VCFs were filtered by Mutect2 FilterMutectCalls module, and FilterByOrientationBias module was used to filter out false-positive calls from OxoG and FFPE. Same as our previous studies [10], the resulting somatic SNVs and indels were further filtered according to the flowing criteria: read depth ≥ 10 in both tumor and normal samples, mapping quality ≥ 40 and base quality ≥ 20, variant allele frequency (VAF) ≥ 5%, and supporting reads ≥ 5 in tumor, VAF in tumor was ≥ 5 times than that of the matched normal VAF. Variants were annotated with Oncotator v1.9.9.0. To further avoid miscalling germline variants at least 19 read depth in the normal sample in dbSNP sites.

Tumor suppressor genes (TSGs) and putative cancer driver genes were obtained from TSGene version 2.0 (https://bioinfo.uth.edu/TSGene/), IntOGen (https://www.intogen.org) database, and COSMIC cancer gene census list (May 2017,http://cancer.sanger.ac.uk/census).

The R package MutationalPatterns [11] (v3.0.1) was used to extract the somatic motifs of these samples. Non-negative matrix factorization (NMF) was used to estimate the optimal number of mutation signatures extracted from WES samples. Cosine similarity were calculated to measure the similarity between our identified signatures and COSMIC signatures v3.2 [cancer.sanger.ac.uk/cosmic/signatures].

### Germline mutation analysis

Germline SNVs and indels were called by GATK HaplotypeCaller. The vcfs were annotated by InterVar [12] v2.0.2 to classify variants based on five-tiered categorization system: pathogenic, likely pathogenic, uncertain significance, likely benign, and benign. Variants were selected if the InterVar or ClinVar annotation matched “Likely_pathogenic” or “Pathogenic”. The possible pathogenic variants in normal samples with read depth ≥ 10, genotype quality ≥ 60, supporting allele reads ≥ 2, and VAF ≥ 0.1 were finally kept.

### Somatic copy number alterations analysis

FACETS (v0.5.14) [13] was used to estimate tumor cellularity and ploidy from paired tumor and normal WES data, and calculated allele-specific somatic copy number alterations. FACETS output was integrated with mutation calls to assign mutation clonality and mutation-specific copy number, including loss of heterozygosity (LOH). Copy number (CN) gains were defined as alterations showing total CN > 2 and CN losses were defined as alterations showing total CN < 2. Arm-level events were defined as any gain or loss occurring in an autosome that involved at least 10% of the arm. Arm-level CNV events with frequency higher than 10% were included to compare the shared proportion between primary and metastatic lesions. To identify significantly focal CNV, we used the GISTIC2 (v2.0.23) [14], which considers both the frequency and amplitude of every CNV, was employed dified parameters “-smallmem 1 -broad 1 -brlen 0.7 -cap 1.5 -conf 0.99 -ta 0.2 -td 0.25—armpeel 1 -genegistic 1 -savegene 1 -gcm extreme -js 4 -maxseg 2000 -qvt 0.25 -rx 0”. The proportion of shared focal CNV events were calculated based on the number of significant focal CNV events. To measure CNV burden, fraction of copy number altered genome was calculated by dividing the number of bases in segments with mean log2 CN ratio > 0.1 or <  − 0.1 by the number of bases in all segments. The average proportion of the genome with aberrant copy number, weighted on each of the 22 autosomal chromosomes, was estimated as the weighted genome instability index (wGII). Large deletion was detected using MPLA kit (SALSA MLPA Probemix P198 FH).

### Tumor clonality and clonal inference

To infer the subclonal population structure for each tumor sample, PyClone-vi (https://github.com/Roth-Lab/pyclone-vi) was used by integrating the variant allele frequencies of each mutation with the FACETS-derived absolute parental copy number information and purity estimates. PyClone generates clusters using a hierarchical Bayesian clustering model. Cluster with less than 3 variants will be removed in following clonal evolution analysis. Copy number changes called by the Battenberg algorithm and read count information of each mutation across all regions in the same tumor were used to calculate cancer cell fraction (CCF). Clonality of mutations was determined based on the Timing analysis using R package “MutationTimeR” [15]. Primary and metastasis shared clonal mutations were defined as Trunk/truncal mutations. According to the methods in a previous study [16], patients with none or very few (< 10) trunk SNVs or diffusely distributed cluster were excluded, which could be probably caused by low tumor purity or low sequencing quality. Clonal ordering and visualization for each patient were reconstructed using R package ClonEvol [17] (version 0.99.11). The number of bootstraps was set as 1000. Minimum probability that a CCF estimate for a clone in a sample is non-negative in an accepted clonal ordering was set as 0.05. Cluster center used median of each cluster. In this study, the most recent common ancestor (MRCA) was defined as the clone/subclone which harbors the full complement of alterations common to all the clones/subclones in the metastatic lesions [16, 18]. In addition, MPTevol was used to analyze the driving role of CNV events in the evolution of FH-RCC [19]. The allele-specific CNA-based phylogenetic trees were constructed based on a minimum event distance for intratumor copy number comparisons (MEDICCs) [20]. The allele-specific CNAs of each sample, obtained from facets, were further filtered by requiring (1) min number of BAF sites 30; (2) max copy number 15; (3) min CNV segment length 1e + 05. (i) number of BAF sites ≥ 30 and (ii) max copy number ≤ 6. The overlapped segments (≥ 106 bp) from all samples and the corresponding numbers of A and B allele were extracted as the input of MEDICC. The bootstrap values of internal nodes were obtained by resampling the distance matrix 1000 times. The constructed phylogenetic trees were further visualized by ggtree (R package, v3.0.1). MEDICC was used to infer CNA-based sample trees, and the phylogenetic tree was finally visualized by MPTevol plotCNAtree function with the number of bootstrap steps 500.

### Infinium MethylationEPIC BeadChip assay

Genomic DNA was treated with bisulfite using the EpiTect Fast Bisulfite Conversion Kits (59,802, Qiagen). All samples were processed in the same batch. Genome-wide DNA methylation profiles of samples (16 primary and 21 metastatic tumors and 8 adjacent normal samples) were generated using Infinium MethylationEPIC BeadChip assay (EPIC array, Illumina). The assay determines DNA methylation levels at > 850,000 CpG sites and provides coverage of CpG islands, RefSeq genes, ENCODE open chromatin, ENCODE transcription factor-binding sites, and FANTOM5 enhancers. The assay was performed according to the manufacturer’s instructions and scanned on an Illumina HiScan. To avoid batch effects, 45 samples were randomly divided into six groups, with 8 samples in a group assayed on the same array.

### EPIC array data processing and DMP identification

Raw EPIC array data were preprocessed using the ChAMP R/Bioconductor package with default settings. Different methylation position (DMPs) analysis was performed on beta (*β*) value. We used a linear model (limma) with the empirical Bayes approach with normal control samples as the reference group. Genomic annotation of CpG sites were annotated using HumanMethylationEPICm probe annotations through ChAMP. The percentage of DMPs in each annotation region were calculated with R software and visualized with ggplot2. A probe was considered significantly differentially methylated if the methylation difference (*β*-values) between the tumor and normal control samples were at least 15% with a FDR-adjusted (Benjamini-Hochberg) *P*-value < 0.01. The methylation of candidate probes for immune genes among different groups was compared using the Kruskal–Wallis test and Dunn’s test. The differential methylated probes between paired samples were also compared using paired *t* test or Wilcoxon signed ranked test. *P*-value < 0.05 was considered as significant difference.

### Methylation profiling analysis

DeepTools2 [21] was used for methylation profiling analysis. In detail, the region between TSS and TES of whole genome genes or specific genes (for example, hallmark gene sets) were normalized into a relative equal length and extending 3000 bp of upstream and downstream. Regions were divided into 50 windows and average methylation was calculated in each window, and then visualized as profile line plot.

### Consensus cluster analysis

As our previous study, consensus cluster analysis was run for FH-deficient RCC cohort alone and combined with TCGA-KIRC/KIRP/KICH cohort, respectively. Methylation β-value matrix was adjusted firstly by removing features with small variance and impute missing value with k-nearest neighbor (KNN). Three feature selection methods (SD, MAD, and CV) and five cluster methods (hclust, kmeans, skmeans, pam, and mclust) were chosen to infer possible stable consensus subgroup from 2 to 6 clusters with different number of top features. The best stable partitions from all methods were chosen based on checking the membership matrix.

### RNA extraction

RNAs were extracted and purified from FFPE tissues by the RNeasy FFPE Kit (73,504, Qiagen, Germany), according to the manufacturer’s instructions, and quantified with Qubit RNA HS Assay Kit (Thermo Fisher Scientific). RNA quality and integrity were characterized using the Bioanalyzer and High Sensitivity RNA ScreenTape (Agilent Technologies).

### RNA-seq libraries

Total RNA was isolated from each sample (29 tumor samples and 5 paired adjacent normal samples) using the Qiagen RNeasy formalin-fixed paraffin-embedded (FFPE) Kit (73,504, Qiagen, Hilden, Germany), following the protocol from the manufacturer. Purity and quantity of total RNA were measured by Nanodrop. Integrity of RNA was evaluated using the RNA Nano6000 Assay Kit on the Bioanalyzer 2100 system (Agilent Technologies, CA, USA). One microgram RNA of per sample was used as input for the RNA sample preparations. Strand-specific RNA sequencing libraries were generated using the Whole RNA-seq Lib Prep kit for Illumina (RK20303, ABclonal, Shanghai, China). Library quality was evaluated on the Agilent Bioanalyzer 2100 system (Agilent, USA). Final libraries were sequenced at the Novogene Bioinformatics Institute (Beijing, China) on an Illumina Hiseq X10 platform by 150 bp paired-end reads.

### RNA sequencing data processing

Raw RNA-Seq reads were trimmed the adapter sequences and filtered low-quality bases using FASTP (v0.20.1) [22], followed by mapping to human genome reference hg19 with STAR (v2.7.9a) [23]. During alignment, STAR was supplied with transcript models GENCODE v19 from https://data.broadinstitute.org/Trinity/CTAT_RESOURCE_LIB/__genome_libs_StarFv1.3. The quality control metrics were obtained using FastQC (v0.11.9) (https://www.bioinformatics.babraham.ac.uk/projects/fastqc/), and alignment quality metrics of bam files were measured using RSeQC (v4.0.0) [24]. RNA abundance was calculated using RSEM (v1.2.28) [25], and the RSEM results were converted with Bioconductor package tximport (v4.1) [26].

### Analysis of differentially expressed genes (DEG)

DEGs were determined using the R package “limma” with cutoff *p*-value < 0.05. For paired/matched lesions from same patient, paired DEGs were calculated using paired *t* test or Wilcoxon signed ranked test. Upregulated genes and downregulated genes were used to perform ontology and pathway enrichment analysis based on Gene Ontology and KEGG databases using R package “ClusterProfiler”.

### Gene set enrichment analysis and single-sample gene set enrichment analysis (ssGSEA)

Gene set enrichment analysis was conducted using the Gene Set Enrichment Analysis (GSEA) software version 4.2.1 [27]. KEGG, GO, wikipathway, and Hallmark gene sets from Msigdb database [28] were utilized. ssGSEA was used for quantifying immune infiltration and activity in tumors using eTME, conserved pan-cancer microenvironment signature, wikipathway, and Immunedeconv signature set. Then, ssGSEA *Z*-score of each signature (i.e., antitumor cytokines) for each sample were used to compare the difference between primary and metastatic lesions. Normalized RNA-Seq data was used as input without further processing (i.e., no standardization or log transformation). Cell cycle score, tumor proliferation score, and immune score were calculated based on ssGSEA analyses. The median ssGSEA score for each subgroup was used in radar chart. For patients who had multiple metastatic lesions, a mean value was used to represent the signature of metastatic tumor. The differential immune-related signature levels between primary and metastatic lesions were grouped according to patients and compared using paired *t* test or Wilcoxon signed ranked test.

### Immunohistochemistry (IHC) and multiple immunofluorescence

IHC and multiple immunofluorescence were performed as previously described [4]. Commercially available primary ki67 (clone MIB-1, 1:100, MXB biotechnologies, Fujian, China) and PD-L1 (clone 22C3, Dako) were used in this study. Multiplex immunofluorescence staining was performed using primary anti-CD163: ab182422 (Abcam, Cambridge, UK); CD20: L26 IR604(Agilent Technologies, California, USA); CD3: A0452 IR503(Agilent Technologies, California, USA); CD4: ab133616 (Abcam, Cambridge, UK); FoxP3: ab20034 (Abcam, Cambridge, UK); CD56: ab75813 (Abcam, Cambridge, UK); CD68:ab213363 (Abcam, Cambridge, UK); CD8: ab178089 (Abcam, Cambridge, UK)); PD-1: D4W2J(86163S, CST, Massachusetts, USA); PD-L1: E1L3N(13684S, CST, Massachusetts, USA): CK: ab7753(Abcam, Cambridge, UK); S100: ab52642 (Abcam, Cambridge, UK). PD-L1 expression was assessed by tumor proportion score, which was defined as the percentage of tumor cells with membranous PD-L1 staining. PD-L1 expression > 1% was defined as positivity. We quantified the numbers of CD3 + T cells, CD4 + T cells, CD20 + B cells, CD68 + /163 + macrophages, and CD68 + /163- macrophages from random five 0.045 mm^2^ fields of lesions. For the quantification of checkpoint molecules, we used CPS and TPS for the assessment of PD-L1 expression and measured the numbers of TIGIT^+^ cells from random five 0.045 mm^2^ fields of lesions. Ki67 index was calculated based on the percentage of Ki67-positive nuclei of tumor cells. This information was described in the “Methods” section.

### Statistics

All comparisons for continuous variables were performed using the two-sided Mann–Whitney test for two groups and the Kruskal–Wallis test for more than two groups. For categorical variables, Pearson’s chi-square test with continuity correction or Fisher’s exact test was used. Pearson correlation analysis or Spearman correlation analysis was used to evaluate the correlation between variables. Survival analyses were conducted using Kaplan–Meier method and the difference was tested using log-rank. A *p-*value less than 0.05 was considered statistically significant.

## Results

### Overview of sample and patient characteristics

In the present study, 19 cases with 23 primary and 35 matched metastatic lesions were selected from our FH-RCC database. To provide the genomic and transcriptomic information of metastatic FH-RCC, we performed whole-exome sequencing in all 58 tumor samples, and Methyl-Seq and RNA-seq were performed in 37 tumor samples and 32 tumor samples, respectively (Additional file 3: Fig. S1A). Baseline clinicopathological characteristics are summarized in Additional file 1: Table S1. The most frequent metastatic sites were retroperitoneal, mediastinal, and cervical lymph node (84.2%, 16/19) and bone (31.6%, 6/19). 17/19 patients received first-line systemic therapies, including tyrosine kinase inhibitor (TKI) monotherapy (41%, 7/17), TKI plus PD-1 inhibitor (47%, 8/17), and TKI plus mTOR inhibitor (12%, 2/17) (Additional file 2: Table S2). Survival analysis demonstrated that patients with PD-1 inhibitor-based therapy achieved more favorable clinical outcomes than those with other therapeutic regimens (objective response rate (ORR) 62.5% vs. 11.1%, *P* = 0.043, median progression-free survival (PFS) 22.7 vs. 9.6mo, *P* = 0.177).

### Comparison of the mutational features between primary and metastatic lesions

FH gene alterations were identified in all included patients, including 8 germline and 11 somatic mutations (Additional file 3: Fig. S1B). All germline and somatic FH mutations were mainly distributed in the lyase domain without hot spot mutation site. Except for case FH07 sharing only one (c.802A > T) of two FH mutations (c.856A > T; c.802A > T), the rest of the primary lesions (*n* = 18) shared the same *FH* gene variants with matched metastatic lesions (*n* = 28). For cases with germline *FH* mutations, additional *FH* gene alterations could be further identified in the second allele, including somatic mutations (FH22, FH38), loss of heterogeneity (LOH) (FH05, FH16, FH21, FH25, FH28), and a large deletion event (FH42). Furthermore, among cases with only somatic FH alterations, three were validated to harbor concurrent FH gene large deletions (FH26, FH29, FH32, Additional file 3: Fig. S1C).

**Fig. 1:**
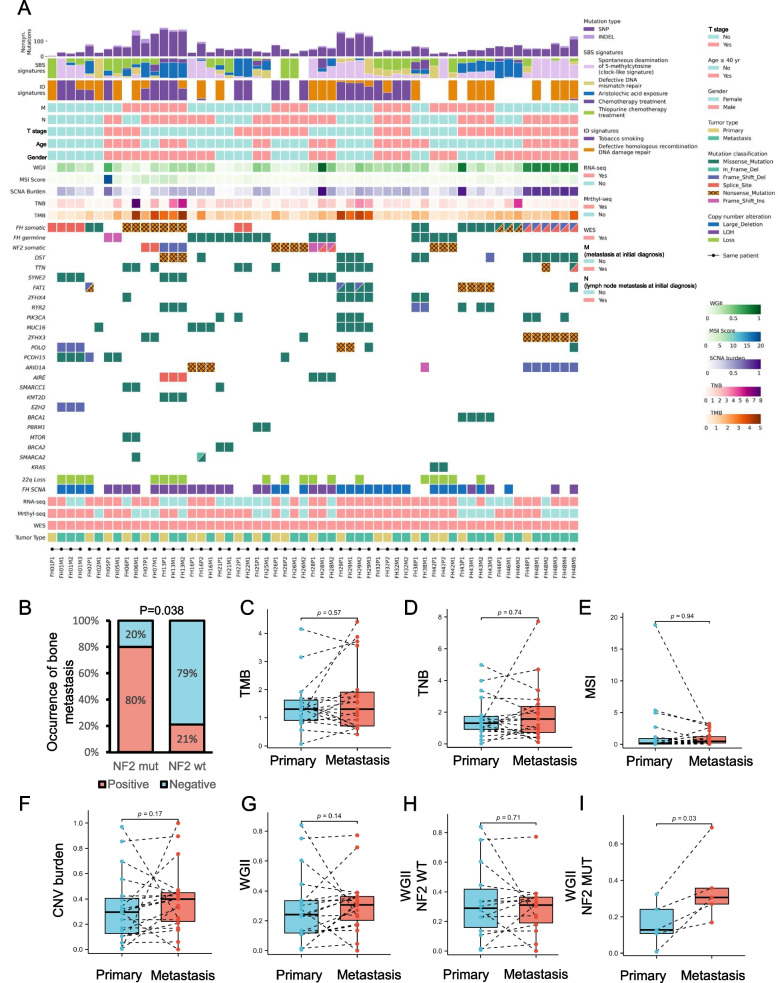
The mutational landscape of paired primary and metastatic lesions of FH-RCC. **A** Integrated genomic and clinical data for 19 primary and matched metastatic lesions. **B** Percentage bar chart of presence of bone metastasis between patients with metastatic NF2 mutation and wild-type NF2. *P*-value was determined by Fisher’s exact test. **C–G** Paired ladder plots depict the changes of TMB, TNB, MSI, CNV burden, and WGII score in patient-matched cases. *P*-value was determined by Wilcoxon signed rank test. **H**,**I** Paired ladder plots depict the changes of WGII score in NF2 wild-type and NF2 mutated subgroups. *P*-value was determined by Wilcoxon signed rank test

Overall, the mutational spectrum of metastatic lesions was similar to that of primary lesions (Fig. 1A). Besides FH, other frequent putative driver mutated genes identified in both metastatic and primary lesions included *NF2*,* DST*,* SYNE2*,* FAT1*,* PIK3CA*,* POLQ*, which were involved in Hippo, PI3K-AKT-mTOR, chromatin remodeling, and DNA damage repair pathways (Fig. 1A). Further analysis revealed cases with *NF2* mutation detected from metastatic lesions were significantly associated with the presence of bone metastasis (80%, 4/5 vs. 21.4%, 3/14, *P* = 0.038, Fig. 1B).

Additionally, we also compared the tumor mutation burden (TMB), tumor neoantigen burden (TNB), microsatellite instability (MSI) status, and mutational signature between primary and metastatic lesions. TMB of metastatic lesions was as low as primary lesions (median 1.31/Mb vs 1.3043/Mb, *P* = 0.57) (Fig. 1C). Both TNB (median 1.31 vs 1.56, *P* = 0.74, Fig. 1D) and MSI score (median 0.19 vs 0.46, *P* = 0.94, Fig. 1E) were similar between primary and metastatic lesions. The SNV-based mutational signature 22, 35, 6, and 87 were consistently common in primary and metastatic lesions (Additional file 4: Table S3).

Metastatic and primary lesions had similar CNV burden (0.40 vs 0.30, *P* = 0.17, Fig. 1F) and weighted genome instability index (WGII) (0.31 vs 0.24, *P* = 0.14, Fig. 1G). In subgroup analyses of WGII, no difference was found between NF2 wild-type metastatic and paired primary lesions (Fig. 1H). Among samples concomitant with NF2 mutation, we found higher WGII in metastatic lesions than primary lesions (median WGII 0.31 vs. 0.13, *P* = 0.03, Fig. 1I). Metastatic lesions shared 59% (16/27) of focal CNV events (Additional file 3: Fig. S2A) and 95% (55/58) of arm-level events with primary lesions (Additional file 3: Fig. S2B). The most frequently shared arm-level events between primary and metastatic tumors included gain of chromosomes 2, 17, 16, 12, and loss of chromosomes 22q, 9, 18, 19, 1 (Additional file 3: Fig. S2C). Notably, metastatic lesions had a higher frequency of 22q loss events than primary lesions (52%, 10/19 vs 21%, 4/19, *P* = 0.046), grouped according to patients (*N* = 19).

### Clonality and phylogenetic trees of FH-RCC

A total of 114 clonal and 61 subclonal mutations were detected in the primary lesion, and 158 clonal and 71 subclonal mutations in the metastatic lesions. We found 68% (78/114) of clonal mutations in the primary lesions remained in the metastatic lesions. Besides, the proportion of shared clonal mutations between the primary and metastatic lesions was statistically higher than that of subclonal mutations (49%, 78/158 vs. 24%, 17/71, *P* = 0.0003). These results indicated that during the metastasis of FH-RCC, metastatic lesions could reserve most primary clonal mutations, and clonal driver mutations may mainly contribute to its metastasis.

**Fig. 2:**
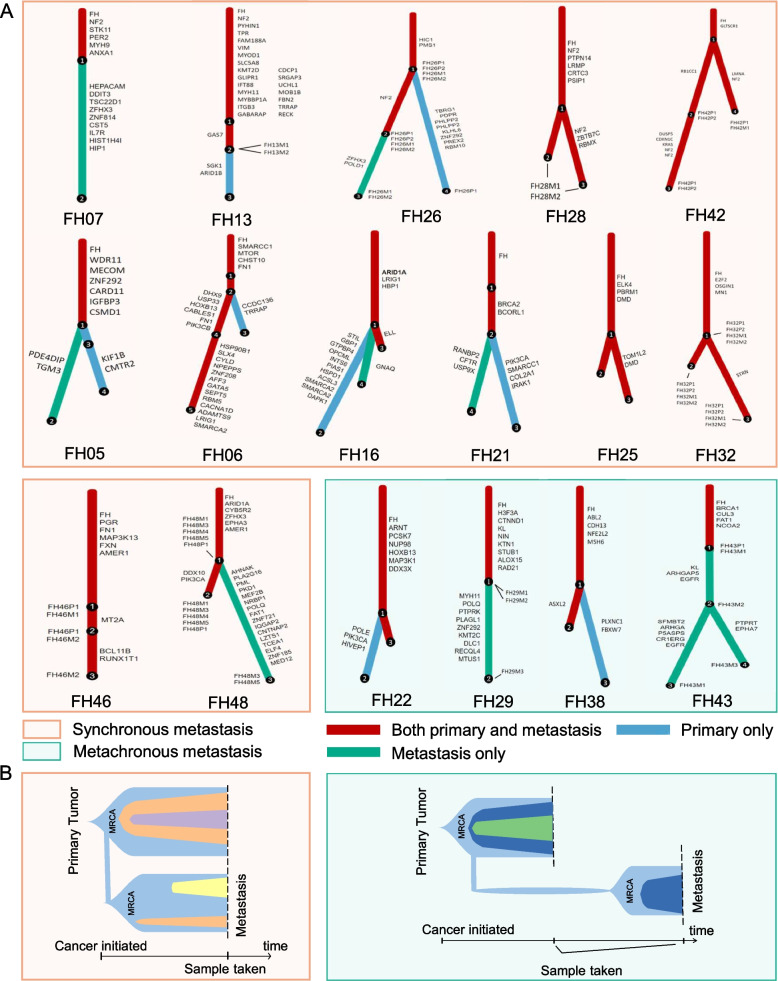
Clonality and phylogenetic trees of FH-RCC. **A** Evolutionary tree and clonal relationship between paired primary and metastasis. Cases were grouped into “synchronous” and “metachronous” metastasis. Metastasis and primary lesions shared clones were marked as red. The most recent common ancestor (MRCA) was denoted by the first node in the phylogenetic tree. **B** Summary of evolution pattern was displayed as fishplots. Patients with synchronous and metachronous metastasis were both featured as MRCA-dominated punctuated evolutionary pattern

Next, we constructed phylogenetic trees in each case using Pyclone and ClonEvol analysis. According to the clonal phylogenetic trees and CNV-based phylogenetic trees, we observed that driver genes (such as FH and NF2) and CNV events (such as 9p loss and 22q loss) were involved in FH-RCC evolution (Fig. 2A, Additional file 3: Fig. S3 and Fig. S4) as previously reported [29]. We defined the most recent common ancestor (MRCA) as the clone or subclone that contains full alterations common to all subclones from metastatic lesions. Based on the clonal phylogenetic trees, we found that all MRCAs of metastatic lesions were uniformly identified as the FH-mutated founding clone of primary lesions, which was demonstrated in both synchronous and metachronous metastases (Fig. 2A and Additional file 3: Fig. S3). Meanwhile, the phylogenetic trees based on the CNAs also confirmed that all MRCAs of metastatic lesions in each case derived from the founding clone of primary lesions (Additional file 3: Fig. S4). Taken together, these results strongly supported that the MRCA of the primary lesion already had the unique rapid metastatic capability and could directly dominate the tumor evolution, suggesting an MRCA-dominated evolutionary trajectory in FH-RCC (Fig. 2B).

### Comparison of transcriptomic features between primary and metastatic lesions

Next, we performed RNA-seq for primary lesions (*N* = 12), metastatic lesions (*N* = 20), and adjacent normal kidney tissues (*n* = 5) from this cohort (Additional file 3: Fig. S1A). Compared with adjacent kidney tissues, both primary and metastatic tissues showed the enrichment of inflammatory response, cell cycle, DNA replication, DNA damage response pathways (Additional file 3: Fig. S5A and Additional file 3: Fig. S5B). Principal component analysis found that for each case, metastatic lesions and paired primary lesions were congregated closely (Additional file 3: Fig. S5C and Additional file 3: Fig. S5D).

**Fig. 3:**
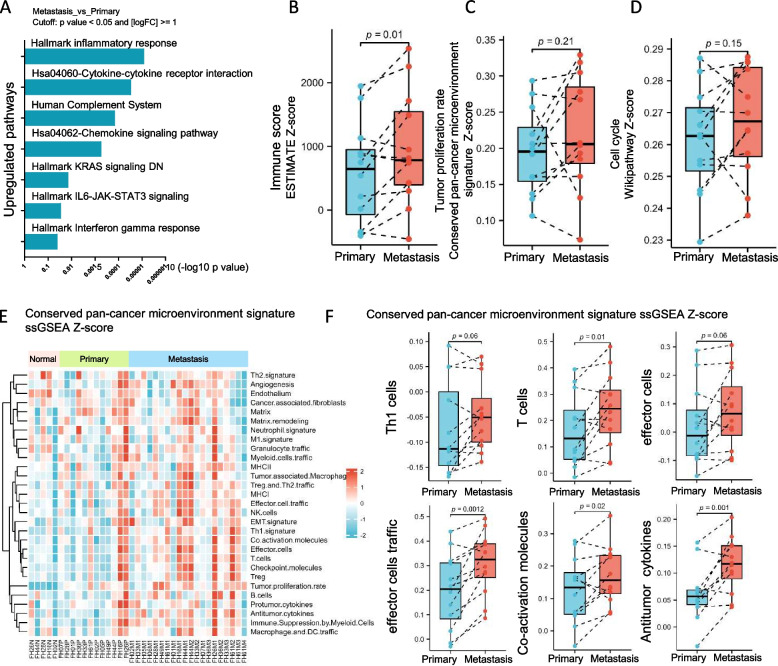
Distinct transcriptomic features and immunogenic phenotype between primary and metastatic lesions in FH-RCC. **A** Enrichment analysis of paired upregulated genes between metastatic lesions and primary lesions. **B** Paired ladder box plots depict the changes of immune score in patient-matched cases. *Y*-axis represents ssGSEA *Z*-score. *P*-value was determined by paired *t* test. **C** Paired ladder box plots depict the changes of tumor proliferation rate in patient-matched cases. *Y*-axis represents ssGSEA *Z*-score.* P*-value was determined by paired *t* test. **D** Paired ladder box plots depict the changes of cell cycle in patient-matched cases. *Y*-axis represents ssGSEA *Z*-score.* P*-value was determined by paired *t* test. **E** Heatmap depicts the conserved pan-cancer microenvironment signature analysis in paired normal, primary, and metastatic lesions. ssGSEA *Z*-score are used. **F** Paired ladder box plots depict the changes of Th1 cells, T cells, effector cells, effector cells traffic, co-activation molecules, and antitumor cytokines in patient-matched cases. *Y*-axis represents ssGSEA *Z*-score. *P*-value was determined by paired *t* test

However, further analysis still found some differentially expressed genes (DEGs) between primary and metastatic lesions. Compared to paired primary lesions, a total of 194 and 510 DEGs were upregulated and downregulated in metastatic lesions, respectively. Importantly, among those upregulated DEGs, immune-related pathways, including cytokine-cytokine receptor interaction, chemokine signaling, inflammatory response, human complement system, IL6-JAK-STAT3, IFN-gamma response, and KRAS DN signaling pathway were predominantly enriched (Fig. 3A). Results from single-sample gene set enrichment analysis (ssGSEA) further showed that metastatic lesions had higher immune scores (median 838.6 vs 644.3, *P* = 0.01, paired *t* test, Fig. 3B), compared to paired primary lesions. But there was no significant difference between metastatic lesions and paired primary lesions in tumor proliferation rate scores (paired *t* test *P* = 0.21, Fig. 3C) and cell cycle scores (*P* = 0.15, paired *t* test, Fig. 3D).

### Higher immune infiltration lymphocytes in metastatic lesions

**Fig. 4:**
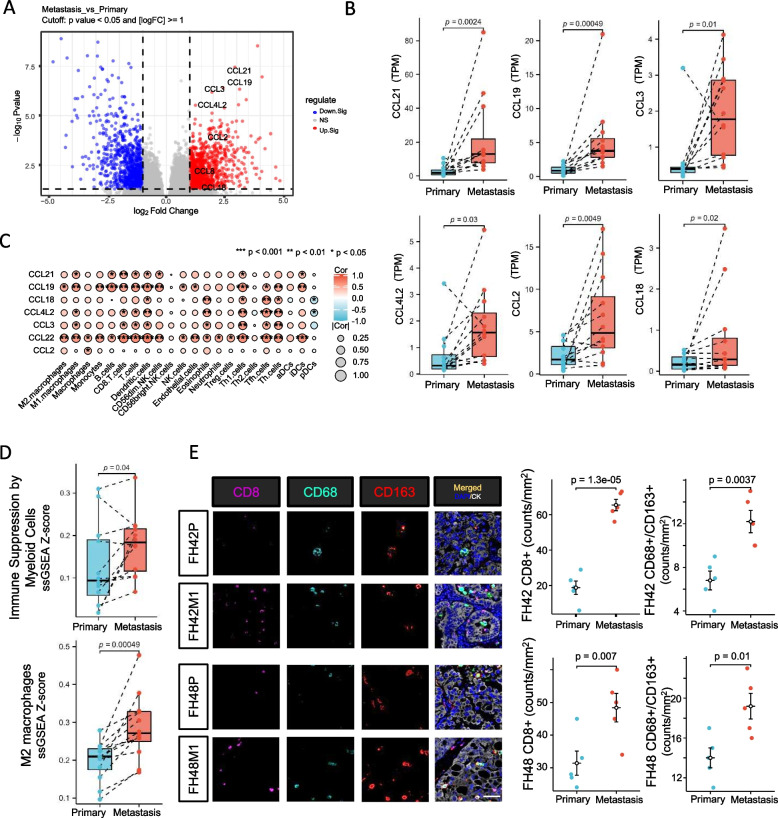
Immune evasion mechanisms of metastatic FH-RCC. **A** Volcano plot of DEGs in metastatic lesions compared with paired primary lesions of FH-RCC. **B** Expression of chemokine ligand family (CCL21, CCL19, CCL2, CCL3, CCL4L2, CCL18) in metastatic lesions, compared to paired primary lesions. *Y*-axis represents TPM of gene expression.* P*-value was determined by Wilcoxon signed rank test. **C** Correlation analysis of chemokine ligand family molecules (TPM) and immune microenvironment signatures(ssGSEA *Z*-score). **D** Paired ladder box plots depict the change of myeloid-derived suppressor cell-related signatures in patient-matched cases. *Y*-axis represents ssGSEA *Z*-score.* P*-value was determined by Spearman correlation analysis. **E** Representative pictures of multiple immunofluorescence staining demonstrated RNA signature-based difference of CD68 + /CD163 + TAMs between primary and metastatic lesions, and quantitative analysis of CD8 + , CD68 + /CD163 + cells in cases FH42 and FH48.* P*-value was determined by *t* test. Scale bar: 100 µm

Based on a conserved pan-cancer microenvironment signature set (Additional file 3: Fig. S5E), both primary and metastatic tumor lesions were identified to have upregulated tumor infiltration lymphocyte (TIL) associated signatures compared to adjacent kidney tissues. Furthermore, compared to primary lesions, matched metastatic lesions showed higher T cells (*P* = 0.01), effector cell traffic (*P* = 0.0012), co-activation molecules (*P* = 0.02) and antitumor cytokine (*P* = 0.001) signatures, and numerically higher Th1 cells (*P* = 0.06) and effector cell (*P* = 0.06) signatures (Fig. 3E, F), indicating the more activated anti-tumor immune-environment in metastatic regions. Multiple immunofluorescence staining of two cases (FH42, FH48) further validated a higher density of CD3 + T cells, CD4 + T cells, and CD20 + B cells in metastatic lesions (Additional file 3: Fig. S6A). We also compared the potential different levels of immune cells between non-lymph node metastases and lymph node metastases, and no difference was found (Additional file 3: Fig. S6B). At the same time, we found much higher expression of chemokine ligand family in metastatic lesions (Fig. 4A, B). Correlation analysis demonstrated a positive correlation of these chemokine ligand molecules with immune cells, such as T cells and Th1 cells (Fig. 4C). These findings suggested the high enrichment level of TILs in metastatic lesions among FH-RCC.

### Immune evasion mechanisms and potential therapeutic targets for metastatic FH-RCC

Despite an activated anti-tumor immune microenvironment within the FH-RCC lesions, we still observed aberrations in several negative immune regulators, which might be involved in immune evasion. We found that myeloid-derived suppressor cell-related signatures, such as M2-like TAMs and immune suppression by myeloid cells, were significantly upregulated in metastatic lesions (Fig. 4D). Multiple immunofluorescence further confirmed the differential levels of CD68 + /CD163 + TAMs between primary and metastatic lesions (Fig. 4E).

**Fig. 5:**
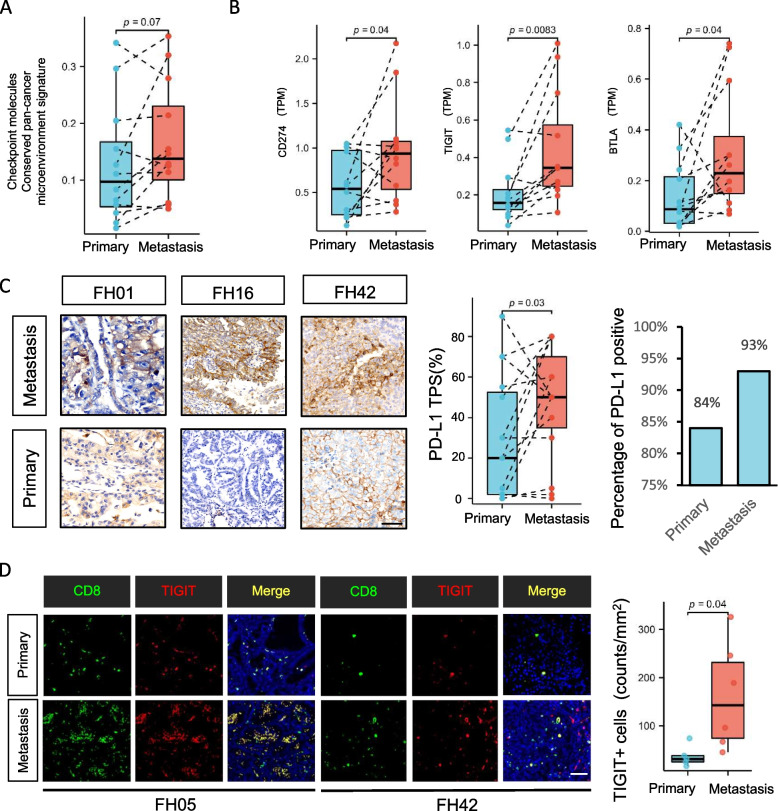
The expression of immune checkpoints in metastatic FH-RCC. **A** Paired ladder box plots depict the checkpoint molecule signatures (measured by ssGSEA analysis) change in patient-matched cases. *Y*-axis represents ssGSEA *Z*-score. *P*-value was determined by paired *t* test. **B** Paired ladder box plots depict the changes of CD274, BTLA, and TIGIT in patient-matched cases. *Y*-axis represents TPM of genes. *P*-value was determined by Wilcoxon signed rank test. **C** Expression and quantitative analysis of PD-L1 by IHC in metastatic and paired primary lesions. Scale bar: 100 µm. **D** Expressions of CD8 and TIGIT were quantitatively analyzed at the protein level by multiple immunofluorescence staining in patient-matched cases.* P*-value was determined by *t* test. Scale bar: 100 µm

In addition, ssGSEA revealed numerically higher checkpoint molecule signatures (Fig. 5A) and higher transcriptomic levels of CD274, BTLA, and TIGIT (Fig. 5B) in metastatic lesions. The expression of PD-L1 and TIGIT were also validated at the protein level (Fig. 5C, D). Both primary and metastatic lesions manifested high expression of PD-L1 (positive rate for primary lesions 84%, metastatic lesions 93%), which was consistent with our previous findings [4] (Fig. 5C). These results might not only explain potential mechanism for immune evasion but also imply promising therapeutic targets for FH-RCC.

### Transcriptomic features between primary and metastatic lesions in FH-RCC harboring NF2 mutation

**Fig. 6:**
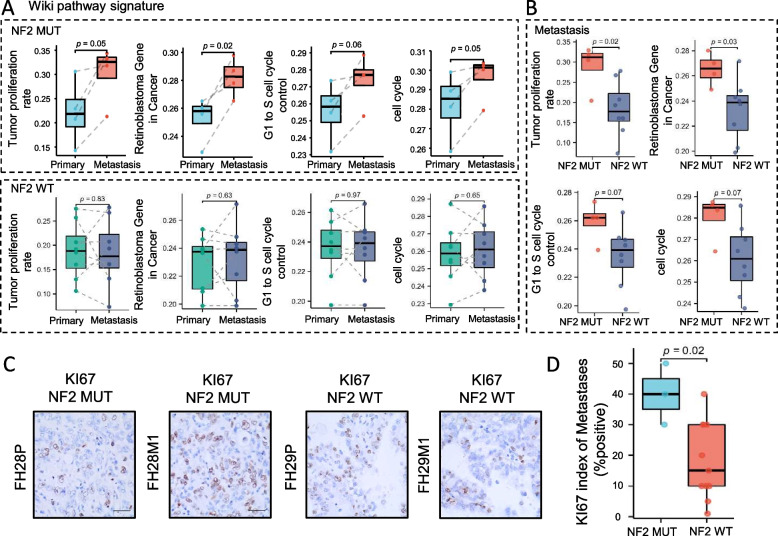
Transcriptomic features between primary and metastatic lesions in FH-RCC harboring NF2 mutation. **A** Paired ladder box plots depict the changes of signatures of tumor proliferation rate, retinoblastoma gene in cancer, G1 to S cell cycle control, and cell cycle in patient-matched cases. The above and inferior panels indicated NF2 mutation and wild-type subgroups, respectively. *P*-value was determined by paired *t* test. **B** The box plot of signatures of tumor proliferation rate, retinoblastoma gene in cancer, G1 to S cell cycle control, and cell cycle in NF2 mutated metastatic lesions compared to NF2 wild-type lesions. **C,D** Representative image of KI67 staining in patient-matched cases (FH28 and FH29), quantitative analysis was performed between NF2 mutated metastatic lesions and NF2 wild-type metastatic lesions using Ki67 index

We also explored the TME features of paired metastatic-primary according to NF2 alteration status. Among cases with NF2 mutation, the metastatic lesions showed obviously higher levels of cell cycle-associated signatures than primary lesions (Fig. 6A) and NF2 wild-type metastatic lesions (Fig. 6B). While no difference was found in cell cycle signatures between metastatic and primary lesions for cases without NF2 mutation (Fig. 6A). Although the relatively small sample size probably impacted this NF2-associated transcriptomic heterogeneity, it still provided some evidence for the vital tumor-shaping role of NF2 alteration in FH-RCC. Ki67 staining results further showed a case that NF2 mutated metastatic lesions had a higher ki67 index than paired primary lesions (Fig. 6C). Among metastatic lesions, cases harboring NF2 mutation had higher ki67 index compared to NF2 wild-type metastatic lesions(*P* = 0.02, Fig. 6D). These results provide initial evidence for the role of NF2 in the evolution of FH-RCC.

### Methylation phenotypes support the activation of immune infiltration status revealed by transcriptome

**Fig. 7:**
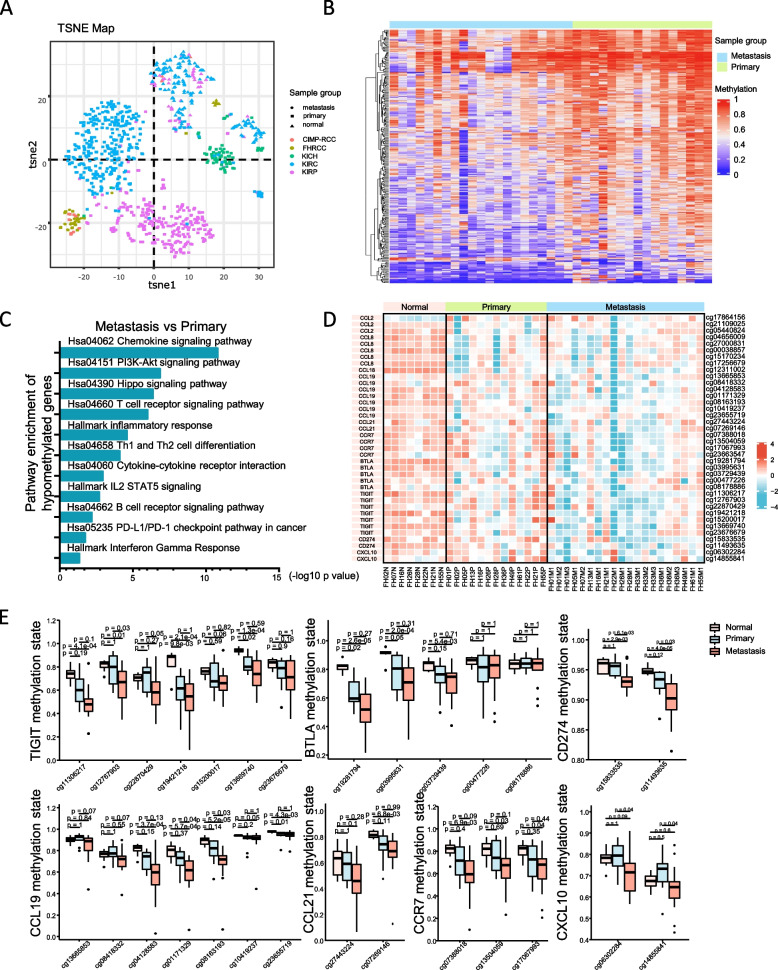
Methylation phenotype and heterogeneity between primary and metastatic lesions. **A** T-distributed stochastic neighbor embedding (TSNE) analysis. Data of FH-RCC included in the present study and TCGA-pan RCC cohort were both integrated into TSNE map. **B** Differentially methylated probes identified between paired metastatic and primary FH-RCC. **C** Enrichment analysis of hypomethylated probes in metastatic lesions (compared to the paired primary lesions). **D** Heatmap of methylation levels of chemokine-related and immune checkpoint-related genes in paired normal, primary, and metastatic lesions. **E** Box plots depict the difference in of hypomethylation degree among normal, primary, and metastatic lesions of FH-RCC. *P*-value was determined by the Kruskal-Wallis test and Dunn’s test

We use EPIC array to compare the methylation pattern of tumor and normal samples. We found that tumors had significantly higher global methylation levels than normal tissues, especially in the upstream area of TSS (transcription start site) and downstream of TES (transcription end site) (Additional file 3: Fig. S7A). TSNE and unsupervised clustering results further verified the different distribution of tumor and normal samples (Additional file 3: Fig. S7B, and S7C). In addition, most metastatic and primary FH-RCC lesions (35/37, 95%) were identified as CIMP phenotype, which was consistent with our previous research work (16/20). We merged 450 K methyl-seq data from TCGA-pan RCC cohort and revealed that all primary and metastatic FH-RCC were distributed into CIMP-RCC cluster through T-distributed stochastic neighbor embedding analysis (Fig. 7A) and unsupervised clustering analysis (Additional file 3: Fig. S7D).

We further compared the differentially methylated probes between primary and metastatic lesions. Although most of the EPIC probes exhibited similarly hypermethylated levels both in primary and metastatic lesions, there was still a small number of probes being identified as differentially methylated probes between paired metastatic and primary FH-RCC lesions, of which only 1305 probes were hypermethylated, and the rest of probes (24,526) were hypomethylated (Fig. 7B, and additional file 3: Fig. S7E).

In line with the transcriptional activation of immune signals, genes with hypomethylated loci were enriched in the inflammatory response, allograft rejection, IL2-STAT5 signaling, and complement pathways (Additional file 3: Fig. S7F and Additional file 3: Fig. S7G) both in primary and metastatic lesions. Compared with paired primary lesions, the hypomethylated probes in metastatic lesions were enriched in genes involved in chemokine signaling, T cell receptor signaling, inflammatory response, Th1 and Th2 cell differentiation, cytokine-cytokine receptor interaction, IL2-STAT5 signaling, B cell receptor signaling pathway, PD-L1 expression, and PD-1 checkpoint pathway in cancer and Interferon-gamma response (Fig. 7C). Specifically, chemokine-related genes (CCL21, CCL19, CCR7) and immune checkpoint-related genes (BTLA, CD274, and TIGIT) were found to have lower methylation levels in both primary and metastatic lesions (Fig. 7D). Further comparison showed that the degree of hypomethylation within the molecules mentioned above in metastatic lesions was more prominent than in primary lesions (Fig. 7E, Additional file 3: Fig. S8A, and S8B).

## Discussion

The present study compared the molecular heterogeneities between metastases and primary lesions of FH-RCC. Our data revealed that FH-RCC was characterized as a unitary MRCA-dominated early evolutionary pattern. Although similarly high levels of lymphocyte infiltration and cell cycle signal were found both in primary and metastatic lesions of FH-RCC, the enrichment of T effector cells and immune-related chemokines, together with immune checkpoint molecules (PD-1/PD-L1, TIGIT, and BTLA), were identified in metastatic lesions. These findings provide the molecular basis for the lethal characteristics and discovery of potential promising therapeutic agents.

The high frequency of early and high-volume distant metastases in FH-RCC is the leading cause of death. Understanding the evolutionary trajectory of metastasis is particularly important for guiding clinical treatment and predicting natural outcomes [15, 29–31]. Based on the analysis of matched primary and metastatic lesions, we found that FH-RCC exhibited an MRCA-dominated punctuated evolutionary pattern. Metastases of FH-RCC could be predominantly seeded directly from the earliest FH-driven MRCA of the primary lesion, which is very similar to a small subset of cases with rapid progression reported by TRACERx renal team [29]. This unique evolution pattern usually suggests that the metastatic competence is acquired in the primary MRCA, leading to rapid progression. It can also explain the feature of high-volume metastases accompanied by small primary lesions, as occult micro-metastases may already be present at the initial diagnosis [29]. Recent studies have revealed that most primary and paired metastatic lesions of RCC are of great distinction, which is consistent with the theory that most RCC evolution are driven by an accumulation of genetic changes (called branched evolution) [29, 32]. In contrast, a minority of RCCs are still found to have low intratumor heterogeneity and rapid progression (called punctuated evolution pattern) [29]. Our genomic analysis found the genomic and transcriptomic aberrations identified in multiple, spatially distinct metastases derived from a single individual were highly concordant, although a limited number of unique events specific to any particular tumor lesion was evident. These findings suggest that clinical decision-making based on a biopsy from a single primary or metastatic site is reasonable.

Different from other RCC subtypes, local treatments for patients with either non-metastatic or metastatic FH-RCC should also be considered for clinical decision-making. Given the MRCA-dominated punctuated evolutionary pattern of FH-RCC, primary tumors may act as a reservoir of metastasis, prompting active local treatments. Hence, local treatment of primary or metastatic lesions appears to be aggressive. This is mainly based on the following aspects. Radical nephrectomy might be the preferred local treatment for those without metastasis to eliminate tumors at the maximum extent. Recent prospective clinical trials (CARMENA and SURTIME) recommended against cytoreductive nephrectomy (CN) for metastatic ccRCC patients [33, 34]. While for patients with metastasis, based on the distinct evolution trajectory, limited genomic heterogeneity between metastatic and primary lesions, together with young age and relatively good performance status, it is reasonable to speculate that patients with metastatic FH-RCC could benefit from CN. Moreover, elevated TILs and PD1/PD-L1 signalings among metastatic lesions could provide additional supportive evidence for CN among patients with metastatic FH-RCC to achieve a more favorable response to PD-1/PD-L1-based immunotherapy. Our FH-RCC database further validated that patients with local treatments have a superior survival benefit than those without local therapies. However, due to the limited cases, selection bias is hard to avoid; we need to take these data seriously and carefully select patients for local treatment. Taken together, these shreds of evidence suggest that active local treatment of primary or metastatic lesions can be applied for suitable cases after careful consideration of the patient’s condition.

Although the transcriptomic characteristics between metastases and primary lesions were generally similar (both with high immunogenicity), some differential findings were still revealed. It has been reported by our and others’ studies that metastatic lesions of RCC have a different immune microenvironment to primary lesions [35, 36]. The metastatic lesions of FH-RCC had echoes of higher immune infiltration than that of the primary lesions, which included enrichment of T effector cells, immune-related chemokines, and upregulation of expression of PD-L1, TIGIT, and BTLA. Furthermore, we found epigenetic changes between primary and metastatic lesions aligned with transcriptomic results. Taken together, these clues could not only give us a reasonable explanation for immune escaping from PD-1/PD-L1 therapy but also imply therapeutic agents targeting these immune checkpoints should be explored in the future (NCT04773951)[37].

Since *NF2* is the most frequently co-mutated gene in FH-RCC, we further analyzed its association with clinicopathologic features. The results showed that NF2 mutation may be associated with bone metastasis. However, little is known about the potential mechanism, further study is needed to validate this finding. In addition, we performed subgroup analysis based on NF2 alteration status and revealed more activated cell cycle signaling in metastatic lesions in cases with NF2 mutation. Cell cycle-related signal has been identified to be involved in PD-1 inhibitor drug resistance in other solid tumors [38–41]. Actually, in clinical results from our FH-RCC database, relatively poor response to PD-1/PD-L1-based therapy and adverse prognosis among patients with NF2 mutation were observed (data not shown). Of course, options such as cell cycle inhibitors are also worth being explored among patients with NF2 mutation. On the other hand, for those without NF2 mutation, cell cycle activation might interfere with PD-1 inhibitor-based immunotherapy, so cell cycle inhibitor combined with PD-1 inhibitor might further improve the efficacy of PD-1 inhibitor-based therapy [42, 43].

Limitations of the present study include the retrospective study design, small sample size, and no fresh tissues. Future multi-center studies with larger sample size and multiple biopsy regions are needed to further verify our findings.

## Conclusions

We identify an FH-mutated founding clone dominated early evolutionary pattern in FH-RCC. More importantly, differences in immune-related signals between metastatic and primary lesions may support potential immune checkpoint-targeting strategy patients with metastatic FH-RCC.

## Supplementary Information

**Additional file 1: Table S1.** Baseline characteristics of patients with FH-deficient RCC.

**Additional file 2: Table S2.** Treatment and molecular information of patients with FH-deficient RCC.

**Additional file 3: Fig. S1.** The sample and mutation information of included FH-RCC cases. **Fig. S2.** Copy number variation of 19 primary-metastatic paired FHRCC cases. **Fig. S3.** Fish plot of each FH-RCC case. **Fig. S4.** CNV-based phylogenetic trees. **Fig. S5.** Transcriptomic features of metastatic and primary FH-RCC. **Fig. S6.** Exploration of TME features of metastatic lesions. **Fig. S7.** Methylation phenotype and heterogeneity between primary and metastatic lesions. **Fig. S8.** Differential methylated probes of immune related genes.

**Additional file 4: Table S3.** SNV-based mutational signatures in primary and metastatic lesions.

## Data Availability

The WES data generated in this paper have been deposited at the National Genomics Data Center (NGDC) Genome Sequence Archive (GSA) database (https://ngdc.cncb.ac.cn/bioproject/browse/PRJCA010235)[44]. BioProject accession: PRJCA010235. The DNA methylation and RNA-seq data reported in this study have been deposited in NGDC OMIX database (OMIX ID: OMIX001275, https://ngdc.cncb.ac.cn/omix/preview/eeP9IvzN; OMIX001280, https://ngdc.cncb.ac.cn/omix/preview/vb8wRit2). WES and DNA methyl-seq data of 7 primary lesions (FH01P, FH02P, FH05P, FH06P, FH07P, FH13P, FH16P) were also used in our previous study [4]. Clear cell renal cell carcinoma, papillary renal cell carcinoma, and chromophobe renal cell carcinoma sequencing data were obtained from the Cancer Genome Atlas (https://portal.gdc.cancer.gov/projects/).
