# Supplementary material for: Genomic and transcriptomic features between primary and paired metastatic fumarate hydratase–deficient renal cell carcinoma
Source: Genome Med. 2023 May 2;15:31. doi: 10.1186/s13073-023-01182-7 (PMC10152735; doi:10.1186/s13073-023-01182-7)
Supplement: Supplementary file 1 — Additional file 1: Table S1. Baseline characteristics of patients with FH-deficient RCC. [file 13073_2023_1182_MOESM1_ESM.docx]

Table S1. Baseline characteristics of patients with FH-deficient RCC

| **Patient Characteristics** | **N(%)** |
| --- | --- |
|  |  |
| **Total number of patients** | 19 |
| **Age at diagnosis, years (Median, range)** | 38(26-71) |
| **Gender** |  |
| Male | 11(57.9%) |
| Female | 8(42.1%) |
| **Patients with skin leiomyoma** | 1(5.3%) |
| **Female patients with uterine leiomyoma** | 4(21.1%) |
| **Primary tumor size, cm (Median, range)** | 7.7(3.0-18.3) |
| **T stage** |  |
| <3 | 9(47.4%) |
| ≥3 | 10(52.6%) |
| **N positive** | 10(52.6%) |
| **Synchronous metastasis** |  |
| Yes | 13(68.4%) |
| No | 6(31.6%) |
| **Histopathological patterns** |  |
| Papillary | 12(63.2%) |
| Tubular | 1(5.3%) |
| Adenoid | 1(5.3%) |
| Solid | 2(10.6%) |
| tubulocystic | 2(10.6%) |
| nested | 1(5.3%) |
| Oncocytic | 0(0%) |
| Unknown | 2(10.6%) |
| **ISUP grade** |  |
| 2 | 0(0%) |
| 3 | 14(73.7%) |
| 4 | 4(21.1%) |
| Unknown | 1(5.3%) |
| **IHC staining** | 18(94.7%) |
| FH |  |
| Deficiency | 17(89.5%) |
| Weak | 1(5.3%) |
| Positive | 1(5.3%) |
| 2SC positive | 19(100%) |
| AKR1B10 positive | 19(100%) |
| **Imaging features** |  |
| Solid | 3(15.8%) |
| Single or less cavity | 3(15.8%) |
| Multilocular | 3(15.8%) |
| Unknown | 10(52.6%) |
| **Nephrectomy** | 19(100%) |
| Partial | 1(5.3%) |
| Radical | 13(68.4%) |
| Cytoreductive | 5(26.3%) |
| **Metastatic sites** |  |
| Lymph node | 16(84.2%) |
| Abdominal | 16(84.2%) |
| Above diaphragm | 3(15.8%) |
| Bone | 6(31.6%) |
| Lung | 2(10.5%) |
| Liver | 2(10.5%) |
| Other | 3(15.8%) |
| **Metastatic sites for sequencing (N=29)** |  |
| **Regional lymph node** |  |
| Retroperitoneal | 16(55.2%) |
| Renal hilus | 5(17.2%) |
| Para-aorta | 2(6.9%) |
| Iliac vessel | 1(3.4%) |
| **Distant lymph node** |  |
| Axilla | 1(3.4%) |
| **Psoas major** | 1(3.4%) |
| **Adrenal** | 1(3.4%) |
| **Lumbar region** | 1(3.4%) |
| **Ovary** | 1(3.4%) |
| **Systemic treatment** |  |
| **1st-line (N=17)** |  |
| PD-1 inhibitor-based | 7(41%) |
| TKI plus mTORi | 8(47%) |
| TKI monotherapy | 2(12%) |
| **2nd-line (N=10)** |  |
| PD-1 inhibitor-based | 8(80%) |
| TKI plus mTORi | 1(10%) |
| mTORi monotherapy | 1(10%) |
| **3rd-line (N=4)** |  |
| PD-1 inhibitor-based | 2(50%) |
| TKI | 1(25%) |
| CDK4/6 inhibitor | 1(25%) |

FH-deficient RCC = Fumarate Hydratase-deficient Renal Cell Carcinoma; ISUP = International Society of Urological Pathology; TKI = tyrosine kinase inhibitor; mTORi = mTOR inhibitor
