# Supplementary material for: Genomic and transcriptomic features between primary and paired metastatic fumarate hydratase–deficient renal cell carcinoma
Source: Genome Med. 2023 May 2;15:31. doi: 10.1186/s13073-023-01182-7 (PMC10152735; doi:10.1186/s13073-023-01182-7)
Supplement: Supplementary file 3 — Additional file 3: Fig. S1. The sample and mutation information of included FH-RCC cases. Fig. S2. Copy number variation of 19 primary-metastatic paired FHRCC cases. Fig. S3. Fish plot of each FH-RCC case. Fig. S4. CNV-based phylogenetic trees. Fig. S5. Transcriptomic features of metastatic and primary FH-RCC. Fig. S6. Exploration of TME features of metastatic lesions. Fig. S7. Methylation phenotype and heterogeneity between primary and metastatic lesions. Fig. S8. Differential methylated probes of immune related genes. [file 13073_2023_1182_MOESM3_ESM.docx]

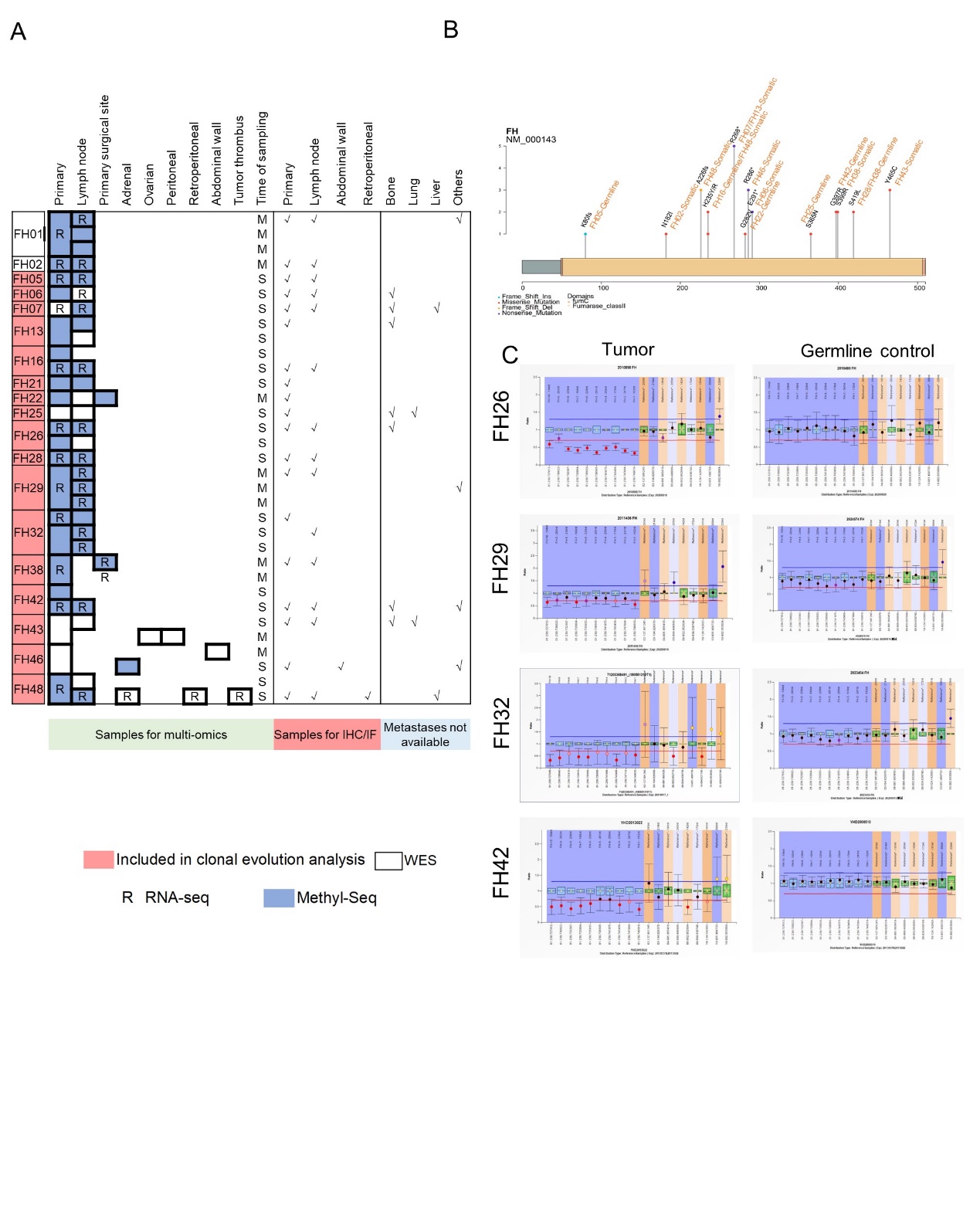
Fig. S1. The sample and mutation information of included FH-RCC cases

1. Samples for whole exome sequencing, Methyl-Seq and RNA-seq were marked with “R”, blue background, and black frame. “R” represents RNA-seq, blue background represents methyl-seq, and black frame represents whole exome sequencing.
2. Mutation site of FH genes in this cohort, germline and somatic were also identified.
3. Large deletion of FH genes.


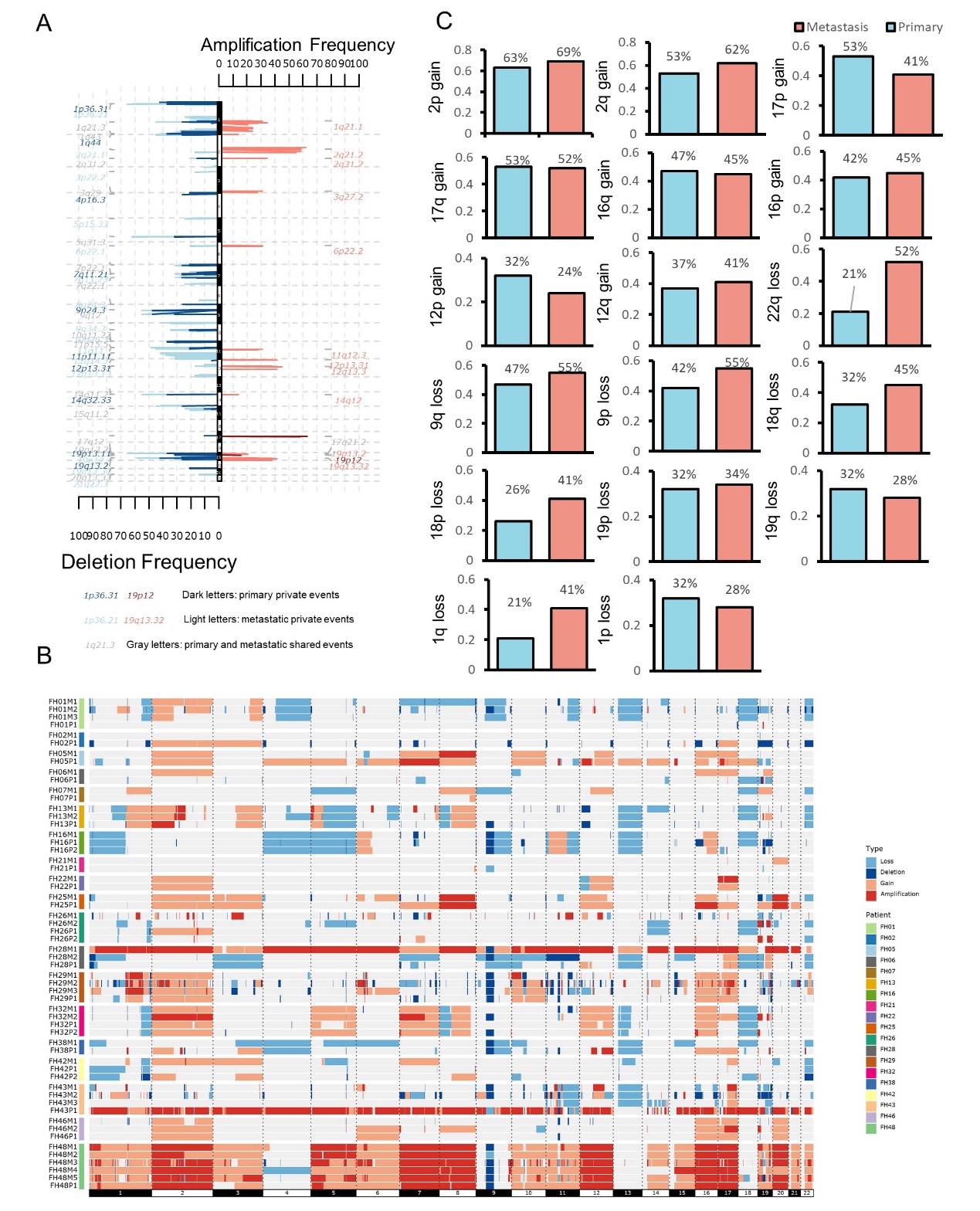


Fig. S2. Copy number variation of 19 primary-metastatic paired FHRCC cases.

1. Focal CNV events in primary lesions and metastatic lesions. Events with q value < 0.25 were included. Gray letters represent primary and metastatic shared events. Dark letters represent primary private events. Light letters represent metastatic private events.
2. Arm-level CNV events map.
3. The frequency of arm-level CNV events in primary and metastatic lesions.


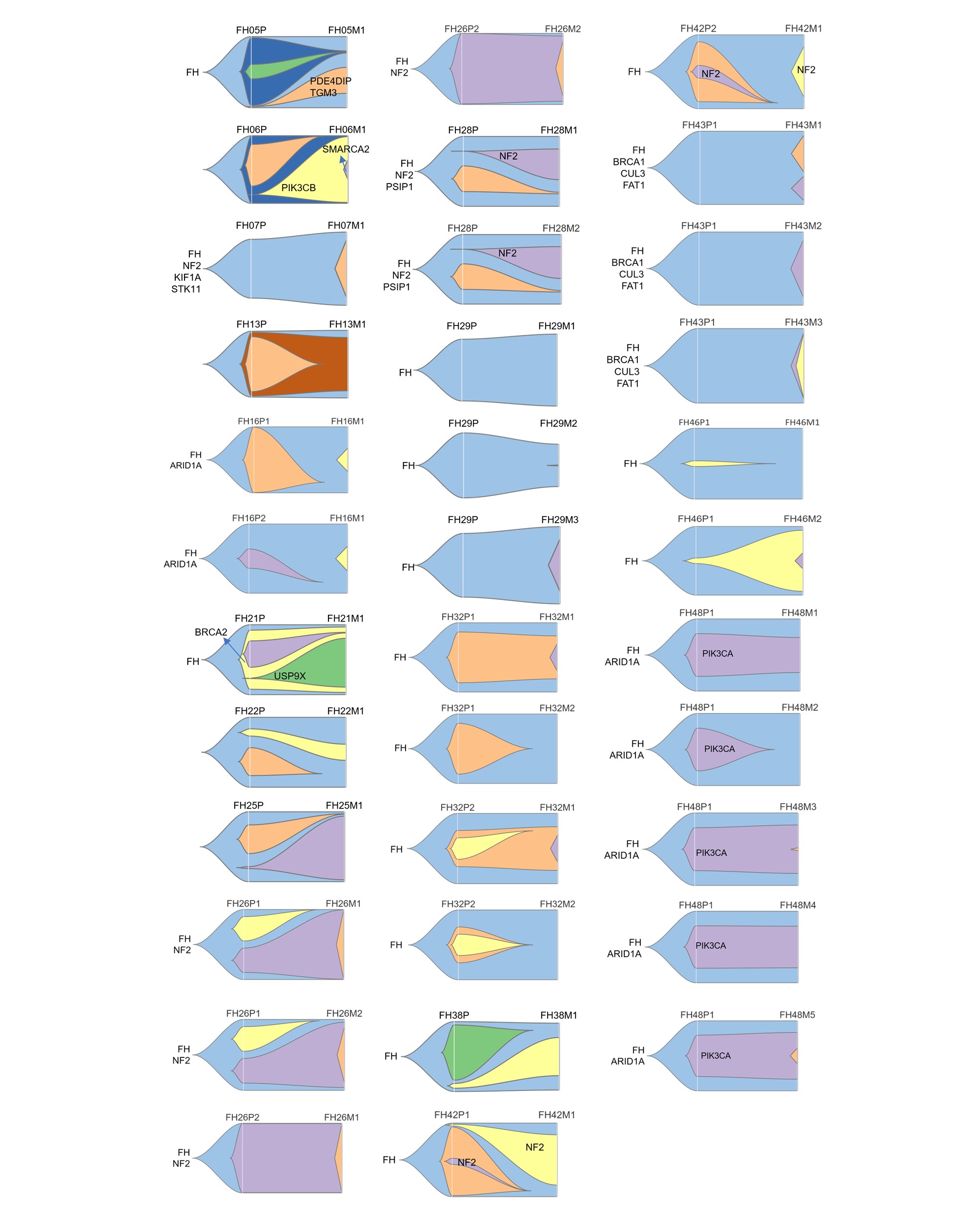


Fig. S3. Fish plot of each FH-RCC case

Each patient’s evolutionary trajectory was shown. White line indicates the clonal composition of primary and metastatic lesions. the most recent common ancestor (MRCA) was defined as the clone/subclone which harbors the full complement of alterations common to all the clones/subclones in the metastatic lesions. MRCA clone was marked in blue. MRCAs of metastatic lesions were identified as the FH-mutated founding clone of primary lesions. Specific driver genes, such as FH, NF2, PBRM1 and so on, were marked.


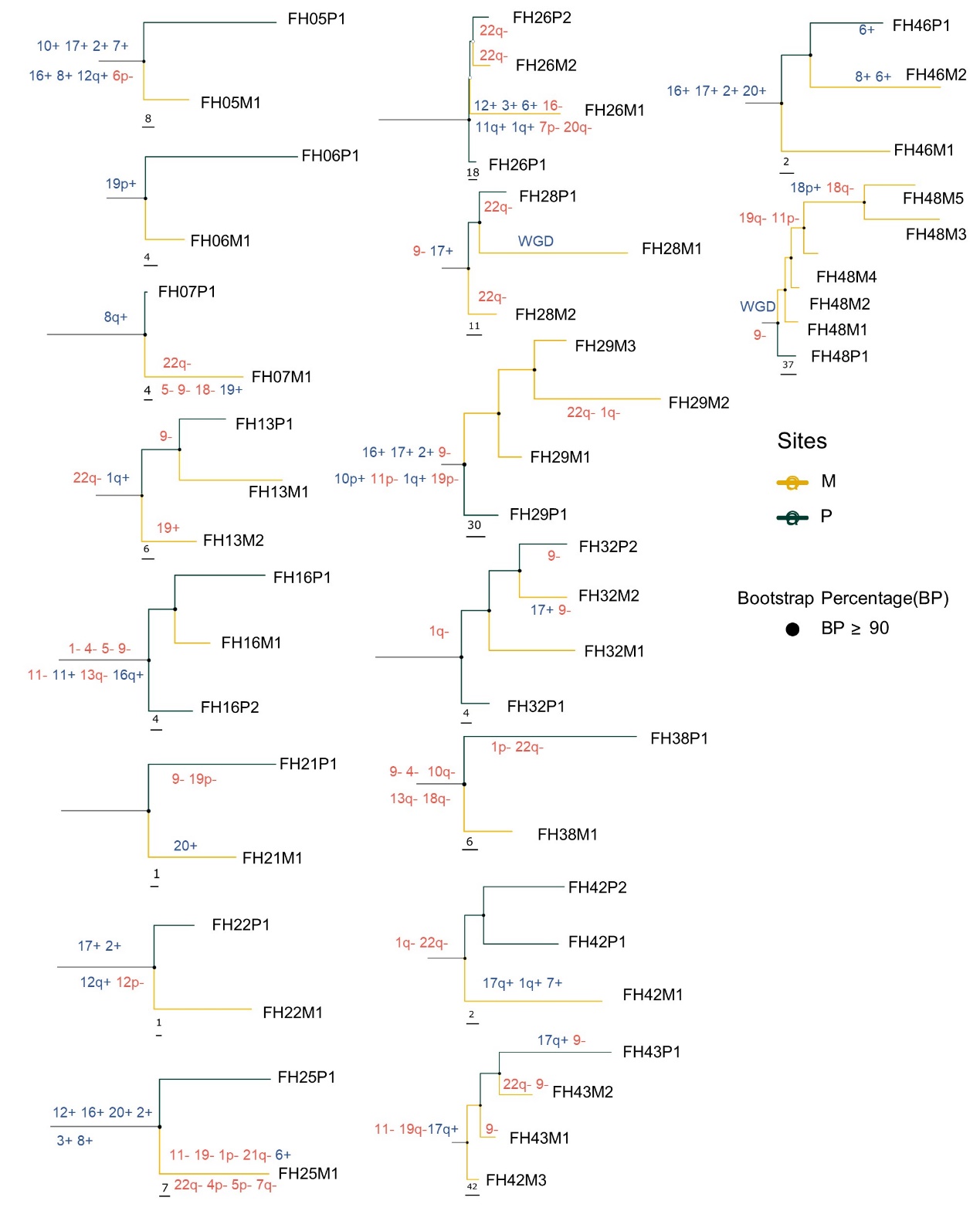


Fig. S4. CNV-based phylogenetic trees

Each patient’s CNV-based evolutionary trajectory was shown. Bootstrap Percentage >90 was labeled using black dot. Yellow line indicates the metastatic lesions and dark line indicated primary lesions. Each scale was labeled according to the number of CNV events. Gain of CNV events were labeled with dark blue letter, and loss of CNV events were labeled with red letter.


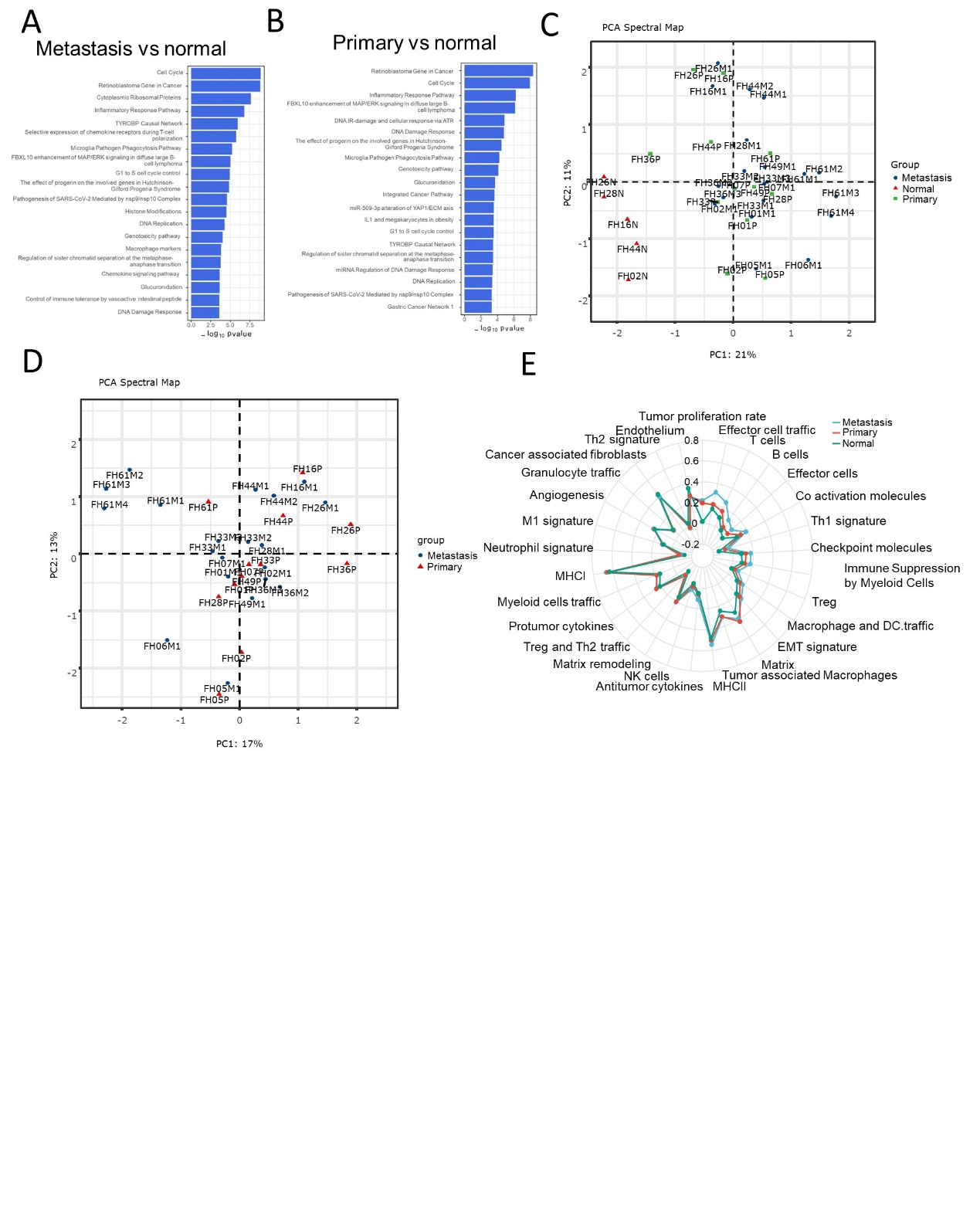


Fig. S5. Transcriptomic features of metastatic and primary FH-RCC

1. KEGG analysis of metastatic lesions compared to adjacent normal kidney tissues.
2. KEGG analysis of primary lesions compared to adjacent normal kidney tissues.
3. PCA analysis of transcriptomic data from metastatic lesions, primary lesions, and adjacent normal kidney tissues.
4. PCA analysis of transcriptomic data from metastatic lesions and primary lesions.
5. Radar plot of TME features (conserved pan-cancer microenvironment signature set) among metastatic lesions, primary lesions, and adjacent normal kidney tissues.


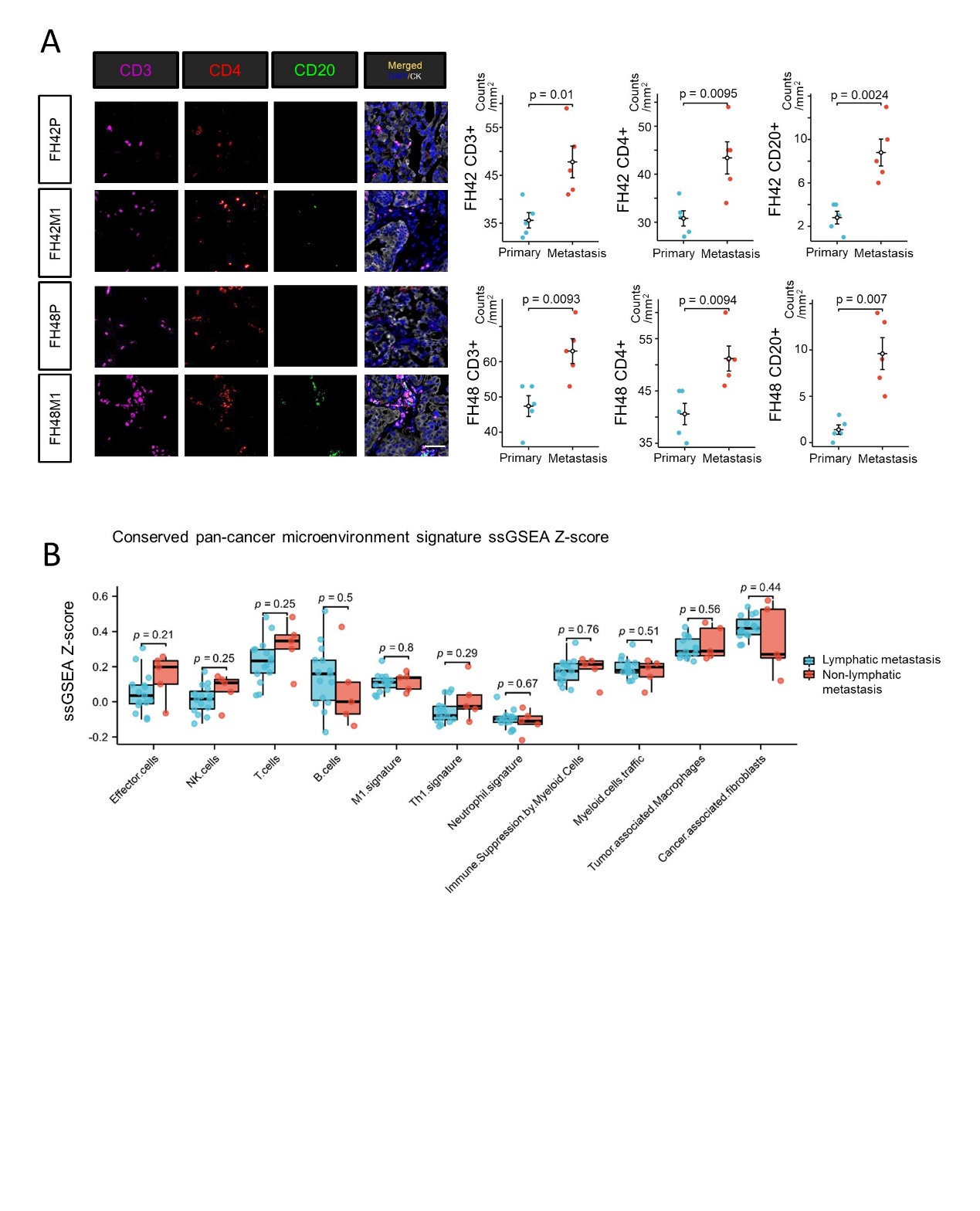


Fig. S6. Exploration of TME features of metastatic lesions

1. Representative pictures of multiple immunofluorescence staining of anti-tumor immune-environment markers (CD3, CD4 and CD20) in paired primary and metastatic lesions (FH42, FH48). quantitative analysis of CD3+, CD4+, and CD20+ cells in cases FH42 and FH48. P-value was determined by t test. Scale bar: 100um.
2. TME features between non-lymph node metastases and lymph node metastases of FH-RCC. ssGSEA Z-score were used. P-value was determined by t test.


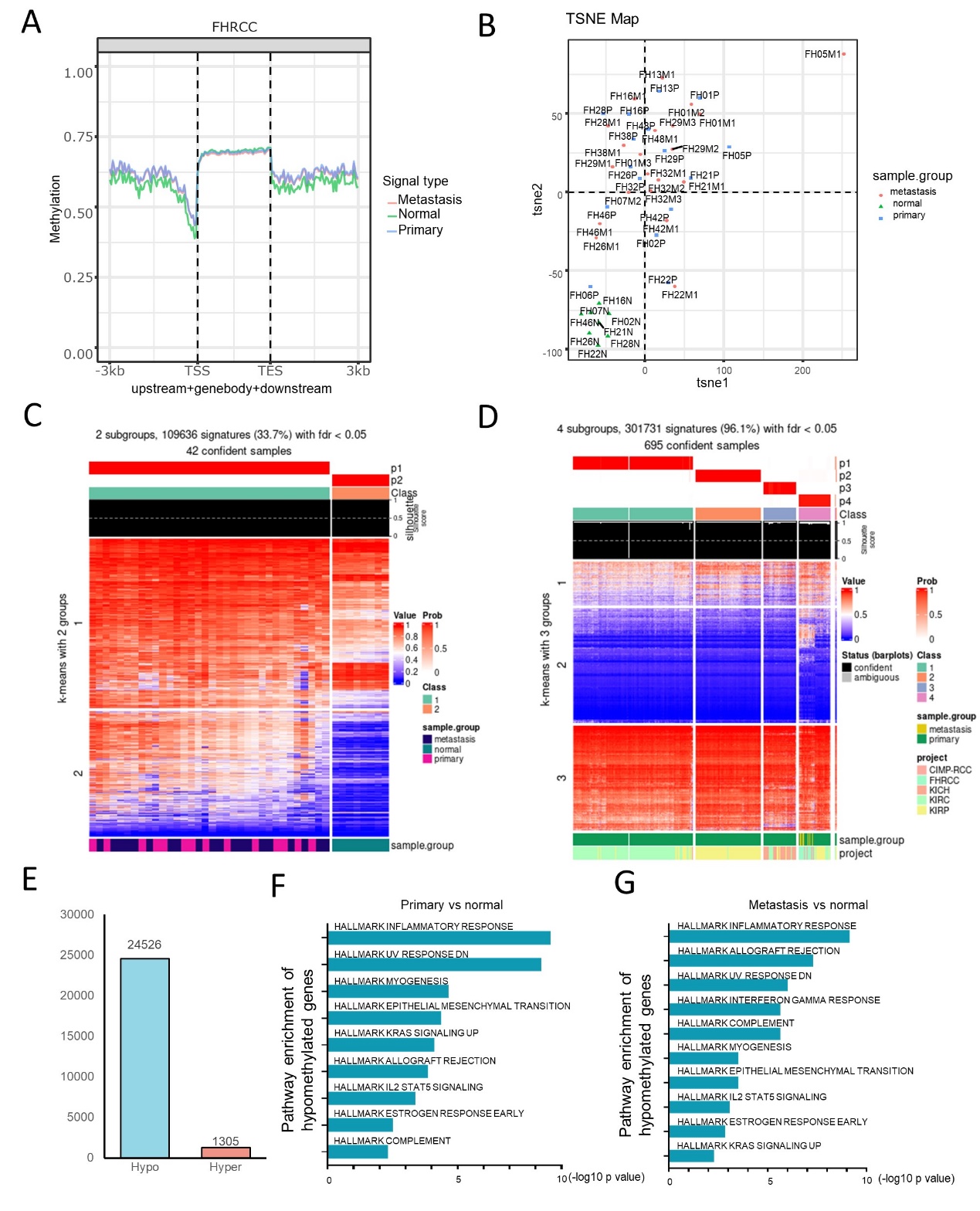


Fig. S7. Methylation phenotype and heterogeneity between primary and metastatic lesions

1. Methylation pattern of metastatic lesions, primary lesions, and adjacent normal kidney tissues.
2. T-distributed stochastic neighbor embedding (TSNE) analysis. Data of primary lesions, metastatic lesions, and adjacent normal kidney tissues were included.
3. Consensus cluster analysis of methylation data from primary lesions, metastatic lesions, and adjacent normal kidney tissues. K-means method was used.
4. Consensus cluster analysis of methylation data from primary lesions and metastatic lesions of FH-RCC, and combined with TCGA-KIRC/KIRP/KICH cohort. K-means method was used.
5. The number of hypermethylated and hypomethylated probes with P<0.05 (paired T test) in metastatic lesions compared to primary lesions.
6. The enrichment analysis of hypomethylated probes-associated genes (primary lesions vs adjacent normal tissues).
7. The enrichment analysis of hypomethylated probes-associated genes (metastatic lesions vs adjacent normal tissues).


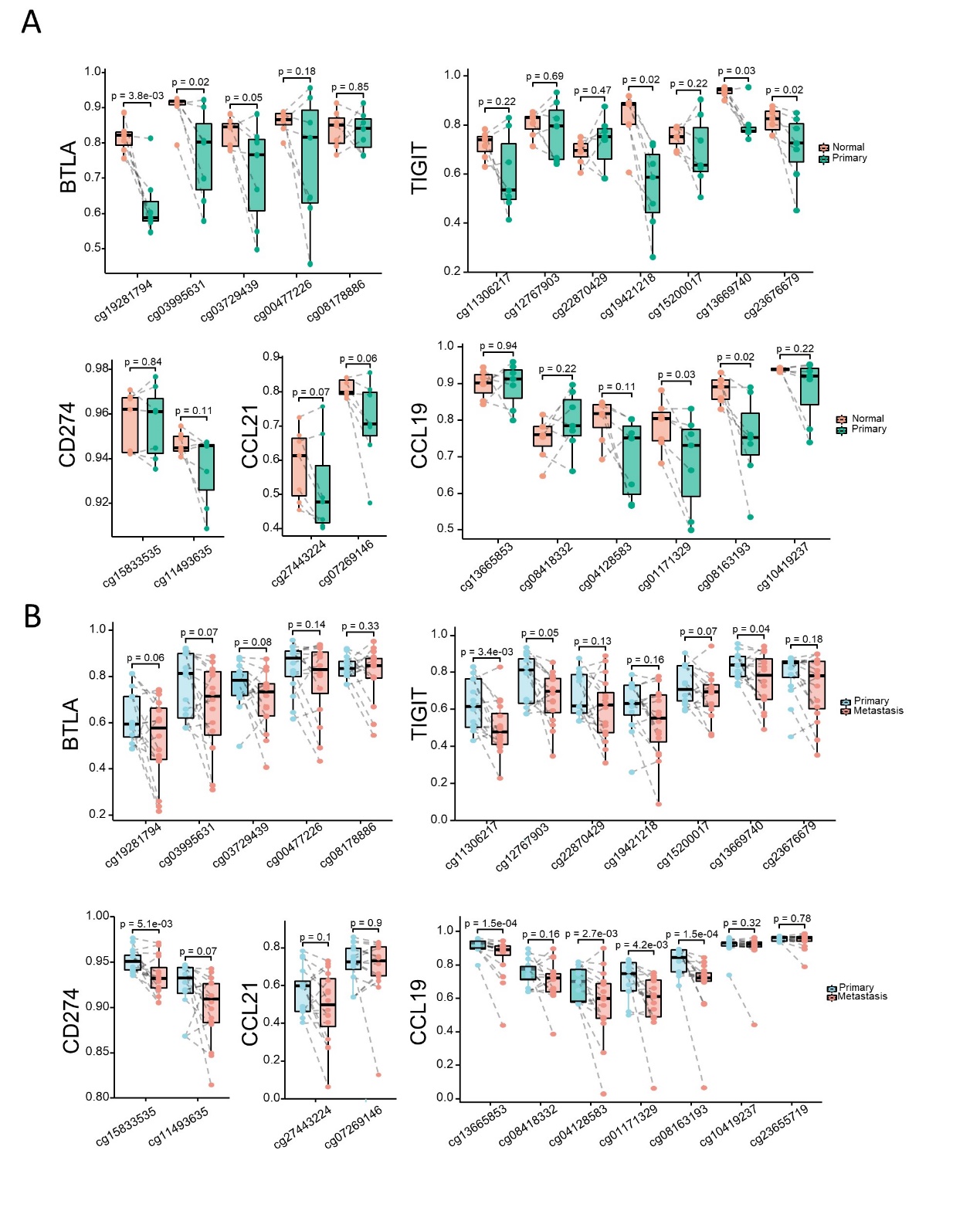


Fig. S8. Differential methylated probes of immune related genes.

1. Paired differential methylated probes between primary lesions and adjacent normal tissues. *P*-value was determined by paired T test.
2. Paired differential methylated probes between metastatic lesions and primary lesions. *P*-value was determined by paired T test.
